# JBP485 promotes corneal epithelial wound healing

**DOI:** 10.1038/srep14776

**Published:** 2015-10-01

**Authors:** Maho Nagata, Takahiro Nakamura, Yuiko Hata, Shumpei Yamaguchi, Taiichi Kaku, Shigeru Kinoshita

**Affiliations:** 1 Department of Ophthalmology, Kyoto Prefectural University of Medicine, Kyoto, Japan; 2 Research Center for Inflammation and Regenerative Medicine, Doshisha University, Kyoto, Japan; 3 Japan Bio Products Co., Ltd., 1-44-4, Tomigaya, Shibuya-ku, Tokyo 151-0063, Japan

## Abstract

Proper wound healing is vital for maintenance of corneal integrity and transparency. Corneal epithelial damage is one of the most frequently observed ocular disorders. Because clinical options are limited, further novel treatments are needed to improve clinical outcomes for this type of disease. In the present study, it was found that placental extract-derived dipeptide (JBP485) significantly increased the proliferation and migration of corneal epithelial cells (CECs). Moreover, JBP485 accelerated corneal epithelial wound healing *in vivo* without inflammation and neovascularization and was found to be effective for the treatment of corneal damage. These data indicate that JBP485 efficiently activates the viability of CECs and has potential as a novel treatment for various kinds of corneal epithelial disease.

The cornea is an avascular and transparent external organ of the visual system composed of three layers: epithelium, stroma and endothelium. The corneal epithelium covers the surface of the cornea as a physical barrier, protecting it by preventing infectious agents from entering the eye. It is constantly regenerated by a reservoir of stem and progenitor cells located in the limbal region^1^, and responds rapidly to heal wounds, using a programmed repair mechanism to immediately close the defect and reestablish its barrier function^2^. The three major cellular events in the re-formation of the corneal epithelium are the proliferation of these cells, the migration of cells from the surrounding basal epithelium to the wounded area and differentiation of the cells into stratified layers. These wound healing processes depend on complex, orchestrated interactions of several growth factors, cytokines and extracellular matrix proteins^3^,^4^.

Hyaluronic acid eye drops or autologous serum (AS) are commonly used to promote wound healing for treatment of corneal epithelial wounds such as superficial punctate keratitis, corneal erosion and persistent epithelial defect^5^. While these eye drops have proved successful in treating intractable corneal wounds, they lack clinical efficiency, and the problems associated with biological characteristics remain^6^. For that reason, further novel treatments with enhanced clinical usefulness are needed to improve the symptoms of these patients.

To develop a novel treatment for corneal epithelial disease, the present work focused on placental extract (PE), which has traditionally been used as a cutaneous wound healer. PE was extensively used in Chinese folk medicine because it was believed to be a rich source of therapeutic components. Nowadays, as the benefits of PE during wound healing have been reported to include anti-inflammatory, anti-fibrotic and anti-oxidative properties^7^,^8^, aqueous PE is available and licensed in many countries for post-surgical dressings, burn injuries and chronic wounds^9^,^10^,^11^, although it can be a potential source of infection because of its human or animal origin.

JBP485 (cyclo-trans-4-L-hydroxyprolyl-L-serine) is a dipeptide that was first isolated from Laennec (a purified hydrolysate from human placenta) as mitogens for a baby hamster kidney cell line^12^. It is noteworthy that JBP485 is completely free from any pathogens, as it can be synthesized by chemical means. Since Laennec has anti-hepatotoxic, anti-inflammatory and anti-apoptotic effects^13^,^14^,^15^, JBP485 has been investigated for these same effects^16^,^17^. However, no study to date has examined JBP485’s wound healing properties.

The purpose of this present study was to investigate the effect of JBP485 on the proliferation, migration and wound healing of corneal epithelial cells. The findings demonstrate for the first time that JBP485 dramatically promotes the proliferation and migration of corneal epithelial cells (CECs); in addition, corneal wound healing was found to be significantly accelerated by JBP485. These data provide new insights into the function of JBP485 in the maintenance of the corneal epithelium.

## Results

### JBP485 accelerates proliferation of CECs *in vitro*

**Figure 1:**
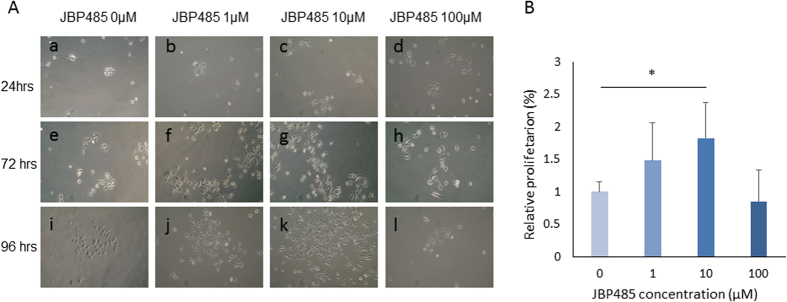
JBP485 accelerates proliferation of cultivated corneal epithelial cells (CECs). (**A**) Representative phase-contrast images of CECs 24 (**a–d**), 72 (**e–h**), and 96 (**i–l**) hours after the addition of JBP485 (**a,e,i**: 0 μM; **b,f,j:** 1 μM; **c,g,k:** 10 μM; **d,h,l:** 100 μM). At 72 hours after the addition of JBP485, higher cell density was observed in plates supplemented with 1 μM (**f**) and 10 μM (**g**) of JBP485 relative to control (**e**). After 96 hours, cell density was noticeably high when CECs were supplemented with 10 μM of JBP485 (**k**) as compared to the others (**I,j,l**). (**B**) BrdU assay at 72 hours after the addition of 0, 1, 10 and 100 μM JBP485, when cells reached subconfluence. Relative proliferation increased with JBP485 in a dose-dependent manner up to 10 μM, with a significant increase observed when CECs were supplemented with 10 μM of JBP485 (p < 0.05). CECs treated with 100 μM JBP485 showed no significant increase. Data are expressed as mean ± SD. *p < 0.05, ANOVA followed by a Dunnett test, n = 5.

To determine the effect of JBP485 on the proliferative activity of CECs, cells were cultured in medium supplemented with various concentrations of JBP485 (Fig. 1A). At 72 hours after the addition of JBP485, a comparatively higher cell density was observed by microscope in those plates supplemented with 1 μM and 10 μM of JBP485 relative to control. After 96 hours, cell density was noticeably higher when CECs were supplemented with 10 μM of JBP485. At the time when cells reached subconfluence, the proliferative activity of each condition was evaluated by 5-bromo-2′-deoxyuridine (BrdU) ELISA assay (Fig. 1B) to support the microscopic findings that showed higher cell densities in the JBP485-treated group than in the control. When CECs were cultivated for 72 hours with JBP485, relative proliferation was found to be increased in a dose-dependent manner up to 10 μM, and a significant increase was observed when CECs were supplemented with 10 μM of JBP485 (p < 0.05, ANOVA followed by a Dunnett test). CECs treated with 100 μM JBP485 showed no significant increase. Relative proliferation value of CECs supplemented with 1 μM and 10 μM of JBP485 was 1.48 and 1.82 respectively (Fig. 1B).

### JBP485 promotes migration of CECs *in vitro*

**Figure 2:**
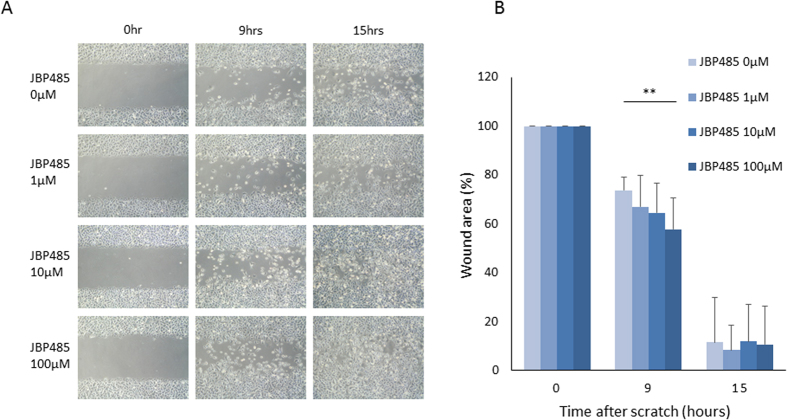
Enhanced migration of CECs by JBP485. (**A**) Cell migration was determined by a scratch assay. Cultivated CECs were scratched linearly. After the scratch, CECs were supplemented with 0, 1, 10 and 100 μM JBP485 and then photographed using phase-contrast microscopy, immediately and at 9 and 15 hours after scratch. (**B**) Chart of averaged wound size change against time. Wound size was significantly smaller than control (no addition of JBP485) when supplemented with 100 μM JBP485 (p < 0.01) at 9 hours after scratch. Data are expressed as mean ± SD. **p < 0.01, ANOVA followed by a Dunnett test, n = 12.

Given the importance of cell migration activity for re-epithelialization, *in vitro* scratch assay of rabbit CECs was then conducted to assess the effects of JBP485 on cell migration. Scratch assay confirmed that JBP485 accelerates closure of the gap in a dose-dependent manner up to a concentration of 100 μM at 9 hours after the scratch (Fig. 2A). A significant difference was observed relative to the control at a concentration of 100 μM (p < 0.01, ANOVA followed by a Dunnett test) at 9 hours after the scratch (Fig. 2B), indicating that JBP485 promotes the migration of CECs *in vitro*.

### Topical administration of JBP485 promotes corneal epithelial wound healing *in vivo*

**Figure 3:**
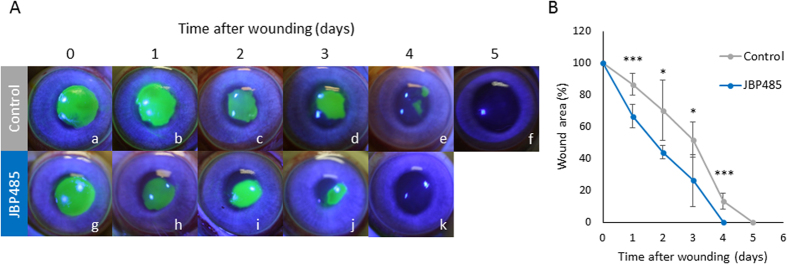
Acceleration of corneal epithelial wound healing by JBP485. (**A**) Representative photographs of the fluorescein-stained corneas of control and JBP485-treated eyes, immediately and 1, 2, 3, 4 and 5 days after wounding. Green-colored areas represent fluorescein-stained regions of the corneal epithelial wounds. In control eyes, corneal wounds needed 5 days to heal completely as compared to 4 days in JBP485-treated eyes. (**B**) Time course of epithelial wound healing in JBP485-treated eyes and controls. Both eyes from 5 mice were used to measure the size of epithelial defects. The wound area was significantly smaller in the JBP485-treated eyes than in control eyes at 1 (p < 0.001), 2 (p < 0.02), 3 (p < 0.02) and 4 (p < 0.001) days. At 5 days, all the wounds were closed in JBP485-treated eyes while wounds still remained in all the control eyes. Data are expressed as mean ± SD. ***p < 0.001, *p < 0.05, Student t-test, n = 5.

Based on the *in vitro* findings, the effect of JBP485 was then examined in an *in vivo* model of corneal epithelial wound healing. An 8-mm corneal epithelial debridement wound was mechanically created in rabbits, followed by topical administration of 100 μM JBP485 or saline to the cornea 4 times a day until the wound was closed. Representative images of healing corneas are shown in Fig. 3A. All the corneal wounds treated with JBP485 closed within 4 days without inflammation and neovascularization while all the wounds to control eyes required 5 days for wound closure. The wound area treated with JBP485 was found to be significantly smaller at 1 (p < 0.001), 2 (p < 0.02), 3 (p < 0.02), and 4 (p < 0.001) days after wounding as compared to control eyes (Fig. 3B).

### JBP485 activates viability of corneal epithelial cells

**Figure 4:**
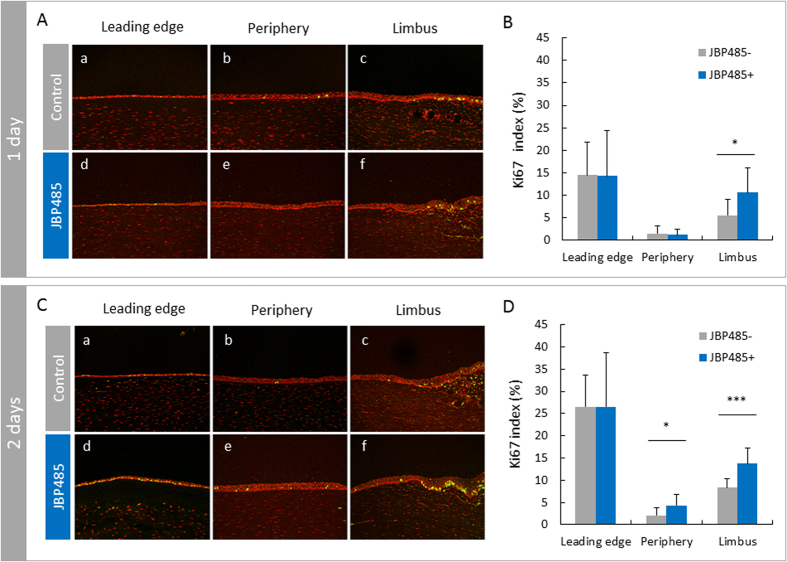
Enhanced proliferation by JBP485 after corneal wounding. (**A**,**C**) Representative images of immunolocalization of Ki67 in the leading edge of migrating epithelium (**a,d**), the peripheral area (**b,e**), and the limbal area (**c,f**) in control (**a–c**) and JBP485-treated corneas (**d–f**) at 1 day (**A**) and 2 days (**C**) after wounding. (**B,D**) Graphs of Ki67 index on control and JBP485-treated eyes at 1 day (**B**) and 2 days (**D**) after wounding. (**B**) At day 1, the Ki67 index of the limbal area was significantly higher in JBP485-treated eyes than in control eyes (p < 0.02). Ki67 indexes of the leading edge and peripheral areas showed no significant differences between groups. (**D**) At day 2, Ki67 indexes of the leading edge area showed high levels in both groups, although no significant difference was found between the groups. Ki67 indexes in the limbal (p < 0.001) and peripheral areas (p < 0.02) were significantly higher in JBP485-treated eyes than in control eyes. Data are expressed as mean ± SD, ***p < 0.001, *p < 0.05, Student t-test, n = 12.

To evaluate the proliferative activity of CECs during wound healing, Ki67 expression was examined for the leading edge, peripheral, and limbal regions of rabbit corneas in each section. Sections were obtained from 12 different parts of each cornea at 1 day and 2 days after wounding. Representative images of Ki67 expression at 1 day (Fig. 4A) and 2 days (Fig. 4C) after wounding revealed a comparatively high density of Ki67 positive cells in the leading edge and limbal areas for both groups (Fig. 4A–C-a,c,d,f). To more precisely compare Ki67 expression between the groups, Ki67 labelling index was calculated. At 1 day after wounding, a significantly higher Ki67 index was observed only in the limbus (p < 0.02) on the JBP485-treated corneas as compared to the control corneas (Fig. 4B). On day 2, each part of those corneas generally showed increased Ki67 indexes as compared to day 1 (Fig. 4D). On day 2, leading edge part of the corneas in both groups demonstrated high Ki67 indexes as compared to other parts, indicating that the leading edge CECs actively proliferated to cover epithelial defects in both groups. On day 2, significant differences between the groups were observed in the limbal (p < 0.001) and peripheral parts (p < 0.02) of the corneas (Fig. 4D). These findings imply that JBP485 efficiently activated the viability of CECs during wound healing even in those areas where proliferative activity of CECs was comparatively low in untreated corneas.

## Discussion

The present study demonstrated for the first time that JBP485, a purified dipeptide from placenta, accelerates corneal wound healing of CECs. When corneal epithelium is wounded, CEC response includes two important steps to cover the corneal surface: cell proliferation from the surrounding area and cell migration to cover the damaged area^3^. In the present study, the effects of JBP485 on cell proliferation and on migration activity were separately evaluated to establish more precisely the mechanism of JBP485. Proliferative activity in cultivated CECs treated with JBP485 at an appropriate concentration was significantly higher than control, which aligns with a previous study in which JBP485 was isolated as mitogens for a baby hamster kidney cell line^12^. In agreement with this study, our results showed that JBP485 has an optimal concentration for cell proliferation. Scratch assay revealed that JBP485 significantly promotes migration in a dose-dependent manner up to 100 μM. These *in vitro* data demonstrate that JBP485 accelerates both proliferation and migration, as evidenced by *in vivo* corneal epithelial wound healing assay, which showed significant acceleration of re-epithelialization when JBP485 was topically administered. In this wound model, the Ki67 index level of the JBP485-treated group was high even in the limbal and peripheral areas that showed a comparatively low Ki67 index level in control corneas. In addition, statistically high significance during early stages of wound healing implied enhanced migration by JBP485, as the healing of the corneal epithelium begins via cell migration to resurface a defect, followed by the proliferation of cells^2^. These results suggest that JBP485 raises the proliferation activity level and the migration level of CECs during wound healing. We posit that, as dose-dependent experiments are ideal for *in vivo* corneal wound experiments (8 mm in size), it is in fact quite difficult to set up their conditions. Therefore, future studies are needed to clarify this point.

Since JBP485 clearly exhibits a liver-protective property^13^, the main purpose of previous studies on JBP485 has been to investigate its anti-hepatitis and liver-protective effects. The present study reports for the first time on its role in wound healing. Those previous studies speculated that the anti-hepatitis and liver-protective mechanism of JBP485 was associated with anti-oxidation and anti-apoptosis^14^. Since these mechanisms are also important for cell proliferation and wound healing^4^, our findings of increased proliferative activity and migration *in vitro* and accelerated wound healing *in vivo* are compatible with previous studies of JBP485.

For treatment of delayed healing of corneal epithelial injuries, many biological substances have been applied in both animal models and human patients. Amniotic membrane has successfully been applied as a temporary biological patch to cover corneal wounds caused by ocular surface diseases such as persistent epithelial defects or infectious keratitis, or as a permanent surgical graft in ocular surface reconstruction, principally after removal of tumors or pterygium^18^. For topical instillation, AS has been applied for treatment of corneal wounds such as severe dry eye, persistent corneal epithelial defects and neurotrophic keratopathy, or for adjuvant use after limbal transplantation or ocular surface reconstruction^6^. These biological substances have successfully improved corneal wounds, but concerns about their human origin still remain. On the other hand, JBP485 also promotes wound healing and without safety concerns as it is chemically defined.

In many studies exploring wound healing in the treatment of persistent epithelial defects, several wound healing factors, including epidermal growth factor (EGF), fibronectin and combination of Substance P (SP) and insulin-like growth factor-1 (IGF-1), have been found to promote wound healing in both animal models and human patients^19^,^20^,^21^. Topical use of EGF has proved successful in accelerating corneal wound closure in animal models and patients by promoting cell proliferation and migration^3^,^19^,^22^. Since biosynthetic human EGF produced by yeast recombinant DNA techniques became available, topical use of EGF has been widely used, mainly in cosmetic applications. However, in the treatment of corneal wounds, corneal angiogenesis is a common complication because the cell proliferative property is related to angiogenesis^23^.

Fibronectin eye drops are also effective for the treatment of corneal epithelial defects because of their ability to accelerate migration of CECs^20^. Additionally, to overcome the potential infection problems associated with the clinical use of blood products, PHSRN—a peptide corresponding to the second cell-binding domain of fibronectin—was developed as an alternative to blood-derived fibronectin eye drops^24^. Its reported beneficial effect was to stimulate migration of CECs in animal models and in clinical studies it was found useful for the treatment of persistent corneal epithelial defects^25^,^26^. The combination of SP and IGF-1 has also been shown to synergistically enhance corneal epithelial wound closure, especially in a rat model of neurotrophic keratopathy^27^. As a developed study of SP plus IGF-1 treatment, when applied to corneal wounds, the tetrapeptide FGLN derived from SP and the tetrapeptide SSSR derived from IGF-1 had successfully promoted corneal epithelial wound healing in both animal models and clinical studies^28^. These fibronectin eye drops, and eye drops containing SP and IGF-1 or their derivatives, aim to exploit its migratory effect. In contrast, topical application of JBP485 was found to promote both proliferation and migration.

For topical application of a wound healer with proliferative effects, sufficient wound healing must be achieved without simultaneous unintended angiogenesis, inflammation or fibrosis. If there is evidence of any adverse effect of the drug, such as angiogenesis caused by EGF eye drops, the drug is less acceptable for clinical use, even where it has sufficient wound healing capacity. In the present study, no signs of adverse effects such as angiogenesis or unintended inflammatory or fibrotic change were microscopically detected in topical application of JBP485 in the animal model. Moreover, the data showed no angiogenesis or inflammatory effect of JBP485 in mouse models or in cultivated CECs (data not shown). In systemic administration, JBP485 has already been shown to have no adverse effects in rats and mice^13^,^14^,^15^. Since JBP485 is thought to be excreted by organic anion transporters in the kidney, concerns might be raised about renal toxicity, but JBP485 is not toxic for the kidney and in fact protects against renal toxicity^29^.

Our recent study showed that JBP485 promotes tear and mucin secretion in corneal and conjunctival cells^30^. Clinically, decreased secretion of tears sometimes makes corneal wound healing slow or intractable. Maintaining the ocular surface in a constantly wet state is thought to be important for proper healing of corneal wounds; when treating corneal wounds from dry eye, JBP485 treatment is therefore of great advantage.

In conclusion, the findings of our investigation show that PE-derived JBP485 efficiently promotes proliferation and migration of CECs. Moreover, JBP485 was found to be effective for the treatment of corneal damage. Although further investigation is needed to elucidate the molecular mechanisms of its biological function, our data provide new insights into the therapeutic potential of JBP485 across a range of corneal epithelial diseases.

## Materials and Methods

### JBP485 preparation

Chemically synthesized JBP485 was provided by Japan Bio Products Co., Ltd. (Tokyo, Japan). JBP485 powder was dissolved in warmed saline (Otsuka, Pharmaceutical Co., Ltd., Tokyo, Japan) into 50 mM solution at a temperature of 50 °C and then diluted to the required concentration, with saline for instillation or with medium for culture.

### Cell culture

Eyes of Japanese white rabbits were purchased from Funakoshi Corporation (Tokyo, Japan). Rabbit corneal epithelial cells were prepared and cultured according to a modified version of the previously reported method^31^. In brief, limbal tissues were removed from the eyes, and the half-layers of endothelial side were then mechanically removed by surgical scissors. The remaining corneal tissues were transferred to minimum essential keratinocyte serum-free medium (KSFM; Life Technologies, California, USA) ([Ca^2+^] = 0.4 mM) containing dispase I (Wako Pure Chemical Industries, Osaka, Japan) at 250 PU/mL and then incubated at 4 °C for 24 hours and 37 °C for 1 hour. The corneal epithelial cells were then peeled and further incubated with TrypLE^TM^ select (Life Technologies, California, USA) at 4 °C for 30 minutes and 37 °C for 5 minutes to dissociate adhesion between the cells. The prepared rabbit CECs were suspended in serum-free CnT-20 medium (CELLnTEC, Santa Cruz, CA, USA) containing 1% Antibiotic-Antimyotic (Life Technologies Corporation, Carlsbad, CA USA) to isolate and grow CECs and then plated in collagen-coated tissue culture plates (AGC Techno Grass Co., Ltd., Shizuoka, Japan). The rabbit CECs were then cultured at 37 °C with 5% CO_2_, and the medium was replaced with fresh medium every 48 hours until the cells reached subconfluence.

### Cell proliferation assay

A colorimetric BrdU cell proliferation ELISA (Amersham Cell Proliferation Biotrak ELISA, GE Healthcare Milan, Italy) was used to assess cell proliferation ability according to manufacturer specifications. Primary rabbit CECs were cultured in serum-free CnT-20 medium containing 1% antibiotic-antimyotic for 3 days until the cells adhered to the culture dish, and then the medium was shifted to the medium containing an appropriate concentration of JPB485 (0, 1, 10, 100 μM) until the cells reached subconfluence. On the day of assay, BrdU (final, 10 μM) was added to each well of a 24-well plate, except for negative controls that received no BrdU. CECs were incubated for 18 hours at 37 °C in a CO_2_ incubator. Plates were drained and blocked with 200 μL/well of ELISA fixative for 30 minutes at room temperature (RT). After decanting, 200 μL of peroxidase-conjugated anti-BrdU solution was added to each well, and dishes were incubated for 90 minutes at RT. Finally, 200 μL of 3,3′,5,5′-tetramethylbenzidine (TMB) solution was added to each well and incubated for 15 minutes on a shaker, and absorbance was immediately measured at 450 nm in an absorption spectrometer (Multiskan MS, Lab systems, Helsinki, Finland).

### *In vitro* scratch assay

*In vitro* wound modelling was performed using a scratch assay. Confluent monolayers of rabbit CECs, cultured as described above in serum-free Cnt-20 medium containing 1% antibiotic-antimyotic in 6-well plates, were linearly scratched with a 1000 μL plastic pipette tip to create a cell-free area. Cultures were gently washed 3 times with warmed phosphate-buffered saline (PBS) to remove loose cells. The CnT-20 medium was then added containing 0, 1, 10 or 100 μM of JBP485, and cells were incubated in a CO_2_ incubator. Twelve fields of each scratch area were photographed at various time points until the cells completely covered the cell-free area. Each wound area was measured using image analyzing software (Image J; developed by Wayne Rasband, National Institutes of Health, Bethesda, MD; provided in the public domain at http://rsb,info.nih.gov/ij). The wound area was calculated as a percentage of the initial wound area.

### Corneal wounding

All experiments were performed in strict accordance with the Association for Research in Vision and Ophthalmology Statement for the Use of Animals in Ophthalmic and Vision Research. All animals used in this study were maintained and handled according to the protocols approved by the Kyoto Prefectural University of Medicine. Albino Japanese rabbits were obtained from Kitayama Laboratories (Kyoto, Japan) and were maintained under pathogen-free conditions in the barrier facility. *In vivo* rabbit models of corneal wound closure were created as previously described^30^. Rabbits were anaesthetised with an intramuscular injection of ketamine hydrochloride (60 mg/kg of body mass; Daiichi Sankyo Propharma, Tokyo, Japan) and xylazine (5 mg/kg; Bayer Medical, Tokyo Japan) as well as with oxybuprocaine eye drop (Santen pharmaceutical company, Osaka, Japan). The central regions of rabbit corneas were demarcated with an 8-mm circular trephine, and epithelia within the circle were mechanically scraped with a rust ring remover (Handy Micro Motor; Inami, Tokyo, Japan). Right eyes were elected to receive treatment while left eyes were assigned to receive the placebo. The right eyes of rabbits received a topical application of 100 μM JBP485, starting immediately after surgery. Control eyes received isotonic saline (Otsuka, Pharmaceutical Co., Ltd., Tokyo, Japan). All eyes were treated 4 times per day for 5 days.

### Corneal Wound Healing Evaluation

The epithelial wounds were visualized using fluorescein and photographed at 0, 1, 2, 3, 4 and 5 days after wounding, under a slit lamp microscope equipped with a digital camera (EOS 30D digital; Canon, Tokyo, Japan) with a cobalt blue filter. The area of each epithelial defect was measured using Image J image analyzing software as above. The wound area was calculated as a percentage of the initial wound area.

### Immunofluorescence

Immunofluorescence analysis was performed by means of the indirect immunofluorescent technique as previously described^32^. Briefly, sectioned samples were immediately washed with PBS and embedded in optimal cutting temperature (OCT) compound. Frozen sections were sliced to a thickness of 8 μm, placed on gelatin-coated slides, air dried and fixed in 100% acetone or 4% paraformaldehyde at 4 °C. After several washings with PBS, the sections were incubated with 1% bovine serum albumin at RT for 30 minutes to block nonspecific binding. They were then incubated with primary antibody (mouse monoclonal anti-Ki67 antibody; BD Biosciences, California) for 1 hour at RT and washed 3 times in PBS containing 0.15% TRITON^TM^ X-100 (The Dow Chemical Company, Midland, MI) for 15 minutes per wash. For negative control experiments, the equivalent serum was used. After staining with the primary antibodies, the sections were incubated at RT for 1 hour with appropriate fluorescein-conjugated secondary antibody. After several washes with PBS, they were coverslipped with mounting medium containing propidium iodide (PI; Vector Laboratories, Inc., Burlingame, CA). The slides were then examined with a confocal microscope (FluoView®; Olympus Corporation, Tokyo, Japan).

### Ki67 labelling index

The sections were stained with Ki67 using the indirect immunofluorescence technique. The Ki67 protein is present during G1, S, G2 and M phases of the cell cycle and is strictly associated with cell proliferation. The epithelial cells positive for Ki67 and PI in a confocal microscopic field were manually counted, and the number of Ki67 positive cells was recorded as a percentage of the total cell count.

### Statistics

Differences were evaluated by analysis of variance (ANOVA) followed by either a Dunnett multiple comparison test or a student’s unpaired t-test. Values of p < 0.05 were considered significant.

## Additional Information

**How to cite this article**: Nagata, M. *et al*. JBP485 promotes corneal epithelial wound healing. *Sci. Rep*. **5**, 14776; doi: 10.1038/srep14776 (2015).
